# HIN6/427: Co-operative Health Information Network in Europe: The Greek experience

**DOI:** 10.2196/jmir.1.suppl1.e37

**Published:** 1999-09-19

**Authors:** E Tambouris, C Makropoulos

**Affiliations:** ^1^NCSR 'Demokritos', AthensGreece

**Keywords:** Community Networks, Health Education, Telemedicine, Greece

## Abstract

**Introduction:**

The co-operative health information network (CHIN) was developed during the last three years in eight regions of six European countries: Finland, Germany, Greece, Spain, Sweden and UK (Scotland). In Greece, the CHIN project is managed by the National Center for Scientific Research 'Demokritos'. The main objective of the CHIN project was to establish a network of regional Co-operative Health Information Networks (CHINs) to support comprehensive and integrated sets of health care telematic services for a broad range of users.

**Methods:**

CHIN provides on-line services for professional and public access. Services for professional access support various working scenarios between hospital staff and doctors in practices outside the hospitals such as remote access to multimedia patient records, quality control for screening results, referrals and resource planning. Services for public access include web-based regional healthcare resource directories (a presentation platform for regional healthcare service providers) on-line consultation and information for health education. Technically, CHIN favourites standardised, simple, open and scaleable solutions for computer and networking technologies (ISDN based Intranets, HTTP) and for the medical applications (DICOM, HL7). Users access the patient records via a standard Web interface.

**Results:**

The Greek resource directory provides bilingual (Greek/English) information that includes: a pilot presentation of a disease (diabetes) focusing on education of children, the largest on-line presentation on the Greek National health system (in Greek only), exclusive lists of all hospitals in Greece, and information on medicine and telemedicine in Greece. Furthermore, two applications have been developed for professionals. The first is an application that runs on a network interconnecting a hospital with a healthcare center and allows the electronic exchange of medical results. The second is the installation and pilot used of a PACS system (DxMM by Medasys) and a Web-based system to access virtual patient records (WebMed by GMD).

**Discussion:**

The Greek CHIN server is one of the largest health-related Web sites in Greece. The ultimate goal is to establish this site as the entry point for Internet users looking for health-related information about Greece. For this purpose, a number of activities have been initiated. For example, collaborations with other health-care related sites, such as the one developed by the ministry of Health and Welfare, are pursued. Also, Web sites are developed and included in the CHIN server free of charge for any Greek hospital and health care center that wishes to participate in this effort. In terms of professional services, the goal in to establish a slide-less hospital, meaning that images of patients from various modalities along with diagnostic reports would be electronically available in all department within a hospital.

